# Antimycobacterial and healing effects of Pranlukast against MTB infection and pathogenesis in a preclinical mouse model of tuberculosis

**DOI:** 10.3389/fimmu.2024.1347045

**Published:** 2024-05-02

**Authors:** Raju S. Rajmani, Avadhesha Surolia

**Affiliations:** ^1^ Molecular Biophysics Unit, Indian Institute of Science, Bengaluru, Karnataka, India; ^2^ Dr. Reddy's Institute of Life Sciences, Hyderabad, Telangana, India

**Keywords:** Mycobacterium tuberculosis (MTB), Pranlukast (PRK), immunomodulation, alveolar macrophages (AMs), interstitial macrophages (IMs)

## Abstract

It is essential to understand the interactions and relationships between *Mycobacterium tuberculosis* (*Mtb*) and macrophages during the infection in order to design host-directed, immunomodulation-dependent therapeutics to control *Mtb*. We had reported previously that ornithine acetyltransferase (MtArgJ), a crucial enzyme of the arginine biosynthesis pathway of *Mtb*, is allosterically inhibited by pranlukast (PRK), which significantly reduces bacterial growth. The present investigation is centered on the immunomodulation in the host by PRK particularly the activation of the host’s immune response to counteract bacterial survival and pathogenicity. Here, we show that PRK decreased the bacterial burden in the lungs by upregulating the population of pro-inflammatory interstitial macrophages (IMs) and reducing the population of *Mtb* susceptible alveolar macrophages (AMs), dendritic cells (DCs), and monocytes (MO). Additionally, we deduce that PRK causes the host macrophages to change their metabolic pathway from fatty acid metabolism to glycolytic metabolism around the log phage of bacterial multiplication. Further, we report that PRK reduced tissue injury by downregulating the Ly6C-positive population of monocytes. Interestingly, PRK treatment improved tissue repair and inflammation resolution by increasing the populations of arginase 1 (Arg-1) and Ym1+Ym2 (chitinase 3-like 3) positive macrophages. In summary, our study found that PRK is useful not only for reducing the tubercular burden but also for promoting the healing of the diseased tissue.

## Introduction

*Mycobacterium tuberculosis* (*Mtb*), the causative agent of tuberculosis is one of the most persistent and lethal pathogens in human history and is still one of the world’s leading causes of death. It infects around 10.0-12.0 million, and kills about 1.6 million people respectively, annually (1, 2). The emergence of drug-resistant strains, co-infection with HIV, and socioeconomic circumstances in developing nations exacerbate further the lethality associated with *Mtb* (3–5). The sobering realization that *Mtb* has become resistant to every frontline medication used to treat tuberculosis highlights the urgent need to develop new treatment regimens and medications (6). Additionally, a six-month-long course of treatment for tuberculosis that includes both first and second lines of treatment with different antibiotics increases the risk of the emergence of drug-tolerant bacteria (7, 8). Therefore, the development of novel medications with the ability to shorten the treatment has become important for the fight against tuberculosis. Nonetheless, several FDA-approved repurposed medications are in various phases of clinical trials to treat tuberculosis infection for reducing the duration that patients must undergo therapy for its treatment (9).

A novel strategy for developing anti-tuberculosis (anti-TB) drugs is to use host-directed methods to target the pathogen’s intracellular survival. Additionally, this approach may be successful in combating drug-resistant strains and reducing the likelihood that new drug-resistant strains will emerge (10, 11). *Mtb* is a facultative intracellular parasite that primarily targets the lung, where it infects the lung resident macrophages as well as other immune cells for pathogenicity, propagation, and dissemination (12). Moreover, macrophages are the most prevalent host cells at infection sites and have been linked to the management and advancement of disease (13). Additionally, macrophages also play important roles in wound healing, homeostasis, tissue integrity, and inflammation regulation (14, 15). The lungs contain two main types of macrophages: tissue-resident alveolar macrophages (AMs) and interstitial macrophages (IMs), which are derived from monocytes. AMs are an M2-type population with anti-inflammatory properties that foster an environment favorable to *Mtb* replication and propagation (16–20). On the other hand, IMs are associated with an immune environment that is more hostile to microbes (21). Hence, comprehending how *Mtb* and macrophages interact is crucial for deciphering the mechanism behind this interaction as well as for the creation of targeted therapies that specifically target the *Mtb*-host interface (11, 22). A number of host factors have been found to support the intracellular survival of Mycobacterium tuberculosis within the macrophage (23, 24). To create targeted therapies for the *Mtb*-host interface and to understand the mechanisms behind the *Mtb*-macrophage interaction, it is crucial to comprehend the interaction and the co-evolutionary relationship between *Mtb* and macrophages (22).

Pranlukast (PRK) is both clinically and commercially available FDA-approved medication for the treatment of asthma (25). Pranlukast, an allosteric inhibitor of MtArgJ (*Mtb* ornithine acetyltransferase) has previously been shown to inhibit the survival and virulence of *Mtb* (26), and by specifically inhibiting the function of cysteinyl leukotriene receptor 1 (CysLT1R), pranlukast possesses anti-inflammatory properties (25).

The goal of the current study was to understand host immunomodulation by PRK better, specifically how Pranlukast (PRK) stimulates the host’s immune system to combat bacterial survival, pathogenicity, and possible host-pathogen interaction. After confirming that PRK has no negative effects on mice’s health and is not harmful to vital organs like the liver, kidney, or lungs, we conducted the infection and treatment experiment to investigate the activity of PRK in acute as well as chronic *Mtb* infection models of mice. Here, we demonstrate that PRK reduced the bacterial burden in the lungs by decreasing the number of *Mtb*-susceptible alveolar macrophages (AMs), dendritic cells (DCs), and monocytes (MO) and increasing the population of pro-inflammatory interstitial macrophages (IMs). Additionally, we surmise that PRK causes the host macrophages to change their metabolic dependence from fatty acid metabolism to glycolytic metabolism during the log phage of bacterial multiplication. Further, we observed that PRK treatment increased the populations of arginase 1 (Arg-1) and Ym1+Ym2 (chitinase 3-like 3) positive macrophage, which in turn improved tissue repair and inflammation resolution. Significantly, we show that Pranlukast (PRK) reduced tissue injury by downregulating Ly6C-positive populations of monocytes. Additionally, within the present study, we found that PRK treatment in *Mtb*-infected mice consistently reduces bacterial burden in the acute model of infection compared to the chronic model, indicating PRK’s bactericidal action. Ultimately, we noted that PRK had a positive impact on tissue pathology repair as well as in tuberculosis load reduction.

## Materials and methods

### Bacterial culture

*Mycobacterium tuberculosis* (H37Rv strain; a virulent laboratory strain of *M. tuberculosis*, American Type Culture Collection) was cultured in Middlebrook 7H9 broth media (Difco) supplemented with 10% albumin-dextrose-catalase (ADC) (Becton, Dickinson), 0.4% glycerol, and 0.05% Tween 80 and the cultures were grown to mid-log phase (OD_600nm_ 0.4–0.7).

### Ethics and animal husbandry

The work plans for the animal experiments were examined and approved by the Indian Institute of Science, Bangalore’s Institute Animals Ethical Committee (IAEC), and the Institute Biosafety Committee (IBSC). The experiment was carried out in compliance with the guidelines set forth by the Committee for the Purpose of Control and Supervision of Experiments on Animals, or CPCSEA. A required number of 4-to 6-week-old female BALB/C mice were procured from the Central Animal Facility at IISc, Bangalore. Before being infected, the animals were allowed to acclimatize for two weeks at the ABSL-3 laboratory.

### *In vivo* toxicity experiments

The toxicity of PRK was assessed in 4-6 weeks-old female BALB/C mice. The mice were divided into two groups (n = 5), one for PRK treatment and the other for PBS treatment as a control. PRK was orally administered @ 40mg/kg/b/wt, weekly at 5 doses for up to 4 weeks, and during this period mice were monitored for health and clinical signs, and weight was taken at weekly intervals for 4 weeks. On the 4^th^ week of post-treatment 500ul of blood was collected from all the animals through the retro-orbital puncture. Before this, the animals were given an inhalation anesthetic treatment consisting of isoflurane (2–3%). Serum was collected from blood and subsequently, blood serum tests were performed to measure the levels of blood urea nitrogen(BUN), serum glutamic pyruvic transaminase (SGPT), and alkaline phosphatase (ALP) level. All animals of both groups were humanely sacrificed by the cervical dislocation method to collect the lungs and liver for histopathological evaluation.

### Study design for the chronic and acute infection models

Chronic model- For the chronic model of infection, BALB/c mice were infected via aerosol through a Madison chamber aerosol generation instrument calibrated to deliver 100 CFU. After aerosol infection mice were housed for establishment of infection for 4 weeks, post which they were randomly grouped (n=6) into “H37Rv Control” and “PRK treated”, and treatment was initiated (PRK; 5 doses weekly@ 40mg/kg.b.wt) through oral route. 8 weeks post-infection, the mice were humanely sacrificed by cervical dislocation method to assess the bacterial load in the lungs.

### Acute model

To assess the bactericidal activity of PRK, we established acute model of infection, in an acute model of Infection, 4-6 weeks female BALB/C mice were infected with 500 bacilli (≈ 500 CFU) of H37Rv, and after one week of post-infection, (≈ 10^4^ CFU), mice were randomly grouped as mentioned in chronic model and treatment started as in chronic models for four weeks (PRK; 5 doses weekly@ 40mg/kg.b.wt through oral route). After that, the mice were humanely sacrificed by the cervical dislocation method to access the bacterial load in the lungs.

### Infection of animals

On the day of infection, the Mid-log phase (OD_600nm_ 0.4–0.7) grown bacterial cultures (H37Rv) were passed through different gauges of needles from lower to higher (23G, 26G, 30G) for making a single cell suspension and turbidity of the culture was measured. Animals were infected through aerosol means using the Madison Aerosol Chamber for Infection. Infected animals (n=5) were sacrificed by the cervical dislocation method, post-24 hours of infection to assess the infection dose. The remaining animals were, humanely sacrificed by cervical dislocation method at the appropriate time point of the study plan. Lungs and spleens were harvested and weighed for assessing CFU. One part of the right upper lobe of the lungs was fixed in 10% neutral buffered formalin for histopathology, the remaining tissues were homogenized in 2ml of PBS and homogenate of lungs in appropriate dilution were plated on 7H11 agar media supplemented with OADC (10% oleic acid, albumin, dextrose, and catalase)and PANTA (polymyxin B, amphotericin B, nalidixic acid, trimethoprim, and azlocillin)for enumeration of CFU. All experiments were conducted in the BSL-3 laboratory of IISC. Bangalore.

### Histological examination and scoring

After sacrificing animals, the right upper lobe of the lung tissues was infused with 10% neutral buffered formalin and preserved until processed for histopathological assessment. At the time of tissue processing, the tissues were embedded in paraffin and sections of 4µm thickness from formalin-fixed and paraffin-embedded tissues were cut onto glass slides and stained with hematoxylin and eosin (H&E) for histopathological evaluation and imaging. The number of granulomas and granuloma areas with specific pathology was assessed.

### Granuloma score

Granuloma scoring was performed by observing and manually counting individual granulomas. we developed a scientific method for scoring the formation of granuloma with specific pathological markers (27). Granulomas with necrosis were given a score of 5, granulomas with no necrosis were given a score of 2.5, and granulomas with fibrosis were given a score of 1. These scores were summed up to provide the total granuloma scores (**Table 1**).

**Table 1:** Methodology for granuloma scoring.

	No. of granuloma w/o necrosis X 2.5	No. of granuloma with necrosis X 5	No. of granuloma with fibrosis X 1	Total granuloma score
**Example**	**5X2.5 = 12.5**	**1x5 = 5**	**0X1 = 0**	**17.5**

### Histopathology scores

The histopathological evaluation was done by the histopathology scoring system for the lung tissues, we developed a scientific method using Mitchison’s virulence scoring system with some modification, considering the number and areas of granulomas, necrosis, alveolar consolidation along with the type of infiltration of immune cells. The histopathology scores were graded as 0-3, (Severe pathology -3; Moderate pathology -2; Minor/minimum pathology -1; No pathology -0) (8, 28).

### Processing of lungs for immune cell isolation

The lungs were removed aseptically, minced, and digested in a 2% FBS/DMEM solution with 0.2 mg/ml liberase and 0.1 mg/ml of DNase (Roche). The mixture was then incubated for 30 minutes at 37°C while being shaken. Subsequently, the pre-digested lung samples underwent processing in a Miltenyi Biotec mild MACS dissociator, following the usual lung dissociation technique. After the lung material was processed, it was put through a 70 µm cell strainer. The filtered samples were then centrifuged at 1600 rpm for 5 minutes at 4°C, and the supernatant was discarded. Next, the pellet containing the red blood cells (RBCs) was lysed with freshly made RBC lysis buffer and allowed to sit at room temperature (RT) for 5 to 10 minutes. To counteract the lysis buffer’s effects, 10 ml of PBS was added to each sample. The lung cell suspensions were then treated for five minutes on ice with Fc block (BD Bioscience), after which the cells were counted and stained for cell surface markers with a cocktail of antibodies and incubated in the dark at room temperature for 30 minutes. For the intracellular antigens, BD Biosciences’ fix and perm buffer were used to fix and permeabilize the cells. The samples were then treated with antibodies in accordance with the BD bioscience protocol. Samples were acquired on a BD FACSAria™ Fusion flow cytometer (BD Biosciences, San Jose, CA). The antibodies used in the present investigation are listed in **Supplementary Table 1**.

### Analysis

The Flow cytometry data were analyzed using BD FACSDiva™ version 8.0.1 and FlowJo 10.8.0 software (BD Biosciences). The gating sequence included the following order: live (SSC-A^+^ FSC-A^+^), FSC-H vs FSC-A to exclude doublets (singlet gate), SSC-A^+^CD45^+^ (leucocytes), and so on.

### Statistical analysis

Statistical analyses were conducted using GraphPad Prism software (version 8.0), and values were presented as mean ± SD. Data information: Statistical significance between experimental groups was determined by a two-tailed, unpaired Student’s t-test (*P < 0.05, **P < 0.01, ***P < 0.001, ****P < 0.0001, and P > 0.5; n.s. not significant).

## Results

This study reports the induction of significant immunomodulation inside the host by Pranlukast (PRK) treatment, resulting in significant sterilization of bacterial burden in both acute and chronic infection animal models of tuberculosis. Further, addresses the potential beneficial effect of PRK on the healing of the lung parenchyma.

### Pranlukast does not exhibit toxicity in mice

**Figure 1:**
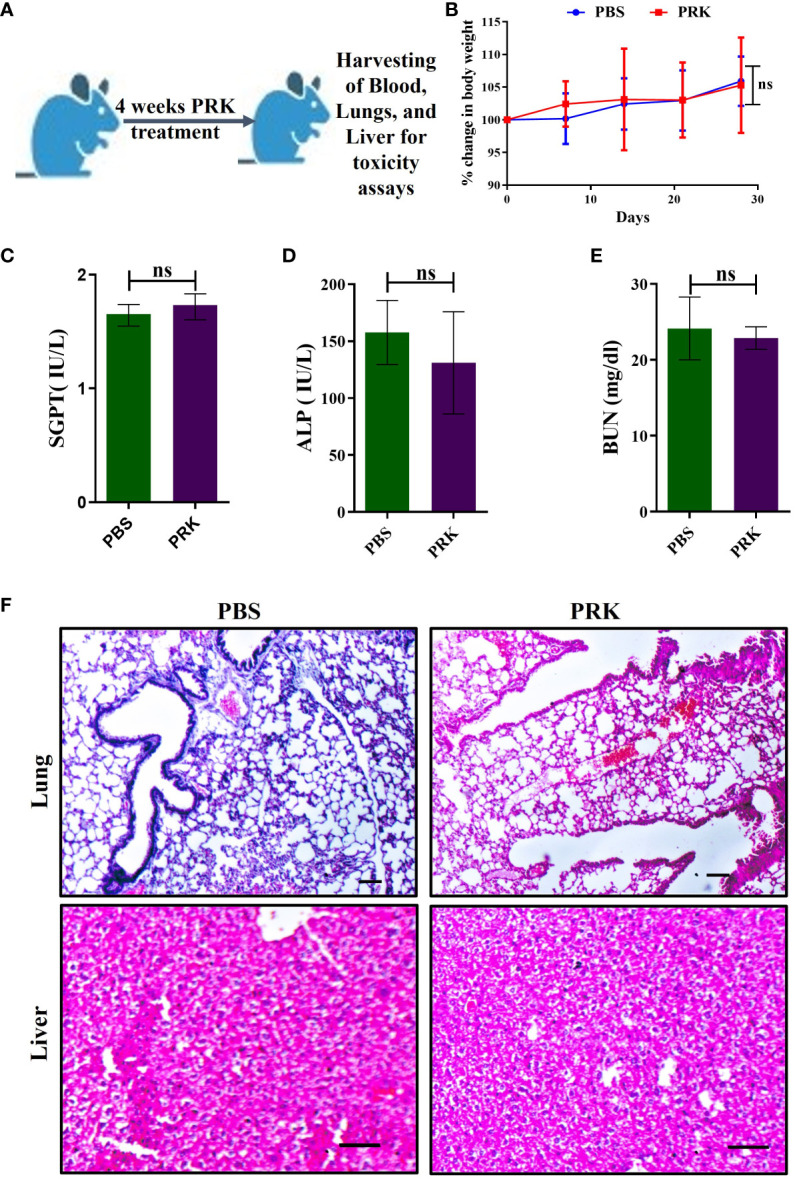
PRK shows no toxicity in mice. **(A)** Schematic of PRK toxicity experiment in mice. **(B)** Percentage of weight gain and loss in PRK-treated and PBS control mice (n=4) of toxicity experiment. Comparative values of SGPT **(C)**, ALP **(D)**, and BUN **(E)** of the PRK treated and PBS control groups, showing non-significant(ns) changes (P > 0.05 calculated by Student’s unpaired t-test). These parameters indicate that the PRK shows no significant hepatotoxic and nephrotoxic effects. **(F)** Representative histopathology (H&E-stained) of the lung and liver of PRK-treated and PBS control groups showed no histological abnormalities upon PRK treatment (Scale bars 50um). ns, non-significant.

To evaluate the toxicity effect of PRK in BALB/c mice, PRK was given orally at a dose of 40 mg/kg/b/wt, five times a week for a maximum of four weeks (**Figure 1A**). Throughout this time, the mice’s health and clinical signs were tracked, and their weight was recorded every week for four weeks. Throughout the whole experimental period, all the cohorts’ animals remained healthy and alive. There were no appreciable variations in body weights between the PBS and PRK groups (**Figure 1B**). During the entire experiment no clinical symptoms such as weight loss or piloerection were seen. Blood urea nitrogen (BUN), serum alkaline phosphatase (ALP), and serum glutamic pyruvic transaminase (SGPT) levels were measured by blood chemistry analysis. Compared to the PBS-treated groups, the enzymatic levels of SGPT and ALP, which serve as indices of liver function, showed insignificant changes in the PRK-treated group (**Figures 1C, D**). Their absolute values (for all animals combined) were mainly contained within the published reference limits (29) suggesting that PRK has no hepatotoxic effects. Insignificant alterations in BUN levels, which are indicators of kidney function, were also observed in all animals in the PRK-treated cohorts compared to the PBS-treated cohort (**Figure 1E**). These findings suggest that PRK did not have any nephrotoxic consequences. Additionally, we found no histological abnormalities in the liver or lungs of the PRK-treated groups (**Figure 1F**), suggesting that PRK is a safe drug for treatment.

### PRK worked better in the acute model of infection and treatment than in the chronic model

**Figure 2:**
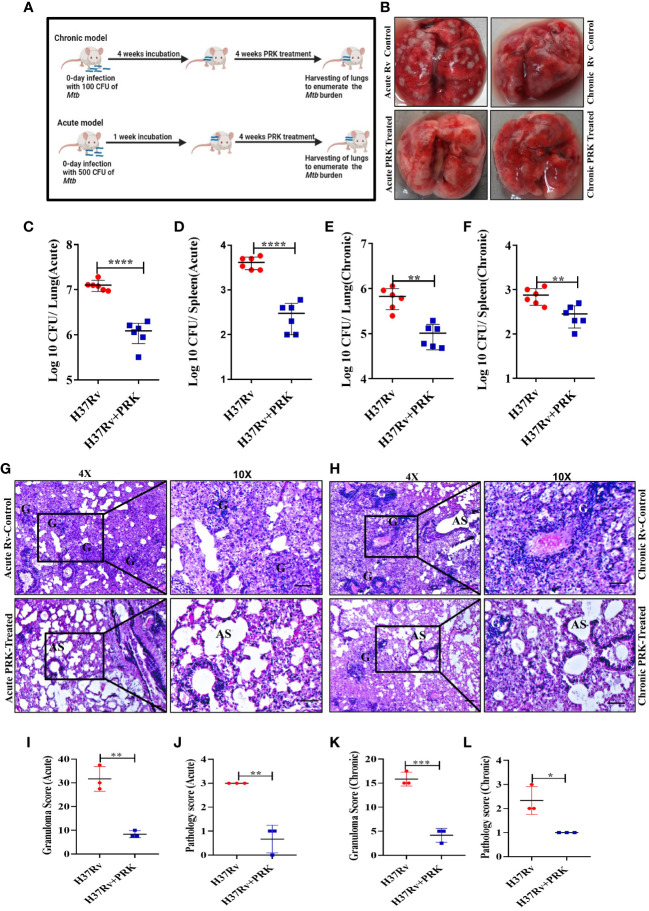
PRK performed better in the acute model of infection and treatment than in the chronic. **(A)** Schematic of *Mtb* infection and drug treatment in BALB/C mice. **(B)** Representative gross morphology of lungs of *Mtb* infected and PRK treated cohort of acute and chronic mice models of infection, the white spot-on lungs correspond to tubercular granulomas. Bacterial count in the lungs **(C)** and spleens **(D)** after treatment with PRK in the acute infection and treatment model of mice. Bacterial count in the lungs **(E)** and spleens **(F)** after treatment with PRK in chronic infection and treatment model of mice. H&E-stained representative images of histopathology of the lungs of the H37Rv infected control and PRK treated in the acute **(G)** and chronic **(H)** model of infection and treatment respectively, G-granuloma and AS represent Alveolar space (Scale bars 50um). Histopathological analysis, granuloma score **(I)**, and histopathology score **(J)** of H37Rv infected and PRK treated groups of acute infection model (n=3). Histopathological analysis, granuloma score **(K)**, and histopathology score **(L)** of H37Rv infected and PRK treated groups of chronic infection model(n=3). Data information: Statistical significance between experimental groups was determined by a two-tailed, unpaired Student’s t-test (*P < 0.05, **P < 0.01, ***P < 0.001, ****P < 0.0001, and n.s. not significant). Mean and standard error (SD) were determined from six biological replicates (n = 6) for CFU and n=3 for histopathological analysis; Bars indicate means ± SD.

After establishing that PRK has no adverse effect on the health of mice. We performed the infection and treatment experiment to check the efficacy of PRK in chronic as well as acute *Mtb* infection models of mice. For the chronic model of infection, 4-6 weeks female BALB/c mice were infected via aerosol through a Madison chamber aerosol generation instrument calibrated to deliver 100 CFU, then mice were housed for 4 weeks of incubation for the establishment of infection, and after 4 weeks of post-infection, the mice were randomly grouped (n=6) as H37Rv Control group and PRK Treatment group, (PRK; 5 doses weekly@ 40mg/kg.b.wt through oral route), and on the 8th week of post-infection (p.i.), the mice were humanely sacrificed for accessing bacterial load in the lungs. For the acute model of infection, 4 -6 weeks female BALB/C mice were infected with 500 bacilli (≈ 500 CFU) of H37Rv through the aerosolization in the Madison aerosol chamber, and after one week of post-infection, mice were randomly grouped as H37Rv infected control and PRK treated group (n=6), and treatment started as in chronic models for four weeks (**Figure 2A** **).** After 4 weeks of treatment in both infection models, the mice were humanely sacrificed to assess bacterial load in the lungs and spleens. In the acute model, the differences in bacterial load between H37Rv control and PRK treated were 1 log, whereas the differences in bacterial load in the chronic model between H37Rv control and PRK treated were 0.8 log in the lungs of mice (**Figures 2C, E** **).** Similarly, the bacterial load between H37Rv control and PRK-treated groups of spleens in the acute infection model was 1.1 logs, whereas the differences in bacterial load in the chronic model between H37Rv control and PRK-treated groups were 0.4 logs. (**Figures 2D, F** **).** The gross pathology (**Figure 2B**) of H37Rv infected control and PRK treated groups in acute and chronic models of infection and treatment, respectively in the lungs were found consistent with the CFU count. We observed severe pathology with multiple small and large granulomas, alveolar consolidation, and infiltration of immune cells in the H37Rv infected control of the acute infection model of lungs (**Figure 2G**). Similarly, we observed severe to moderate pathology in the H37Rv-infected chronic model of mice lungs (**Figure 2H** **).** In PRK treatment groups, in both acute and chronic models of infection, we observed minimum pathology with large areas with normal alveolar space (**Figures 2G, H** **).** Granuloma scores and histopathology scores were observed to be consistent with bacterial infection and PRK treatment in the acute H37Rv control and acute PRK treated groups (**Figure 2I, J**) as well as the chronic H37Rv control and chronic PRK treated groups (**Figures 2K, L**), respectively. Thus, PRK works better in the acute model of bacterial infection than in the chronic model of infection in reducing the bacterial burden. Taken together, these results indicate the bactericidal properties of PRK. The results of acute and chronic infection model are now summarized in **Table 2**.

**Table 2:** Colony forming unit (CFU) in chronic and acute model.

Infection and treatment model	Reduction in bacterial burden in the lungs of PRK-treated vs. untreated groups	Significance	Reduction in bacterial burden in the spleens of PRK-treated vs. untreated groups	Significance
Chronic	0.8 log	**P < 0.01	0.4 log	**P < 0.01
Acute	1 log	****P < 0.0001	1.1 log	****P < 0.0001

### PRK treatment reduced alveolar macrophages, monocytes, and dendritic cells population

**Figure 3:**
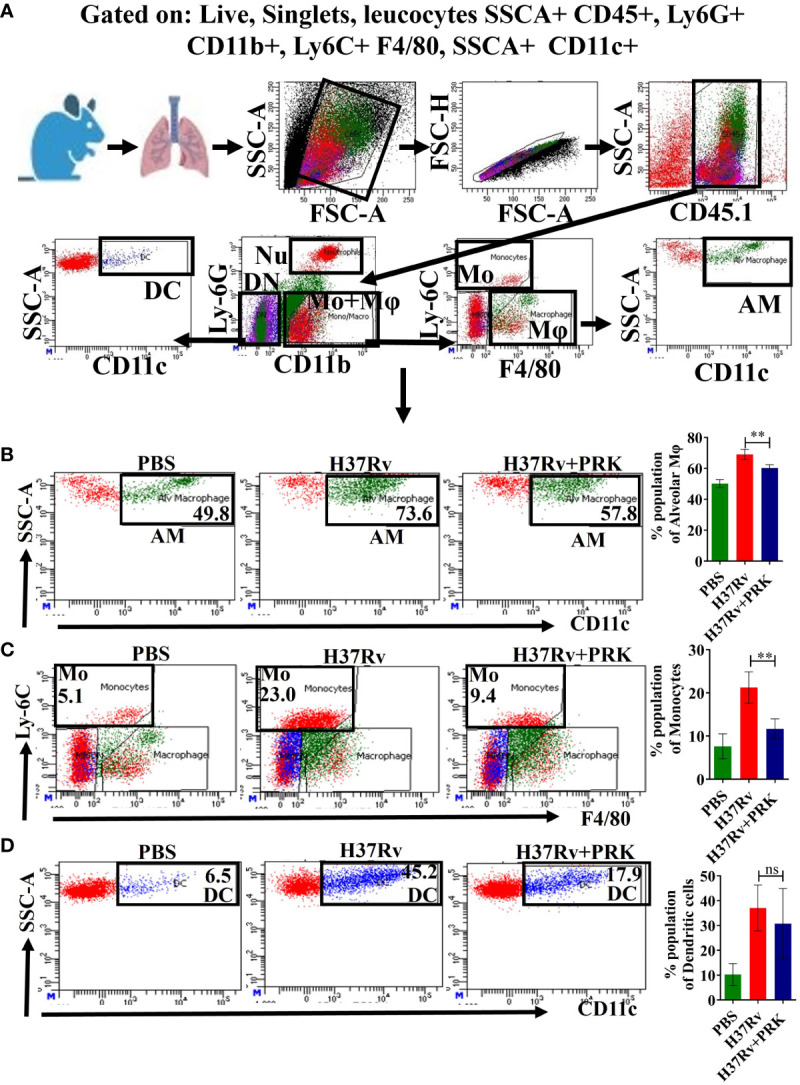
PRK treatment significantly reduced *Mtb* permissive alveolar macrophages (AMs), and monocytes (Mo) populations in an acute model of infection and treatment. **(A)** General gating strategy used for Flow cytometry analysis of the lung immune cells obtained from PBS Control, H37Rv infected, and H37Rv+PRK treated cohorts of mice (n=4). Identification of lung alveolar macrophages (AMs; SSC-A^+^CD11C^+^) **(B)**, monocytes (Mo; Ly-6C^+^ F4/80^+^) **(C)**, and dendritic cells (DCs; SSC-A+CD11C^+^) **(D)** of the acute infection and treatment model of mice (harvesting lungs 5 weeks post-infection), showing representative FACS dot plot with the percentage populations (% of parent cells acquired) of AMs, Mo, and DCs, respectively. Statistical significance between experimental groups was determined by a two-tailed, unpaired Student’s t-test (**P < 0.01, and n.s. not significant). Mean and standard error (SD) determined from four biological replicates (n = 4) for FACS analysis; Bars indicate means ± SD.

**Figure 4:**
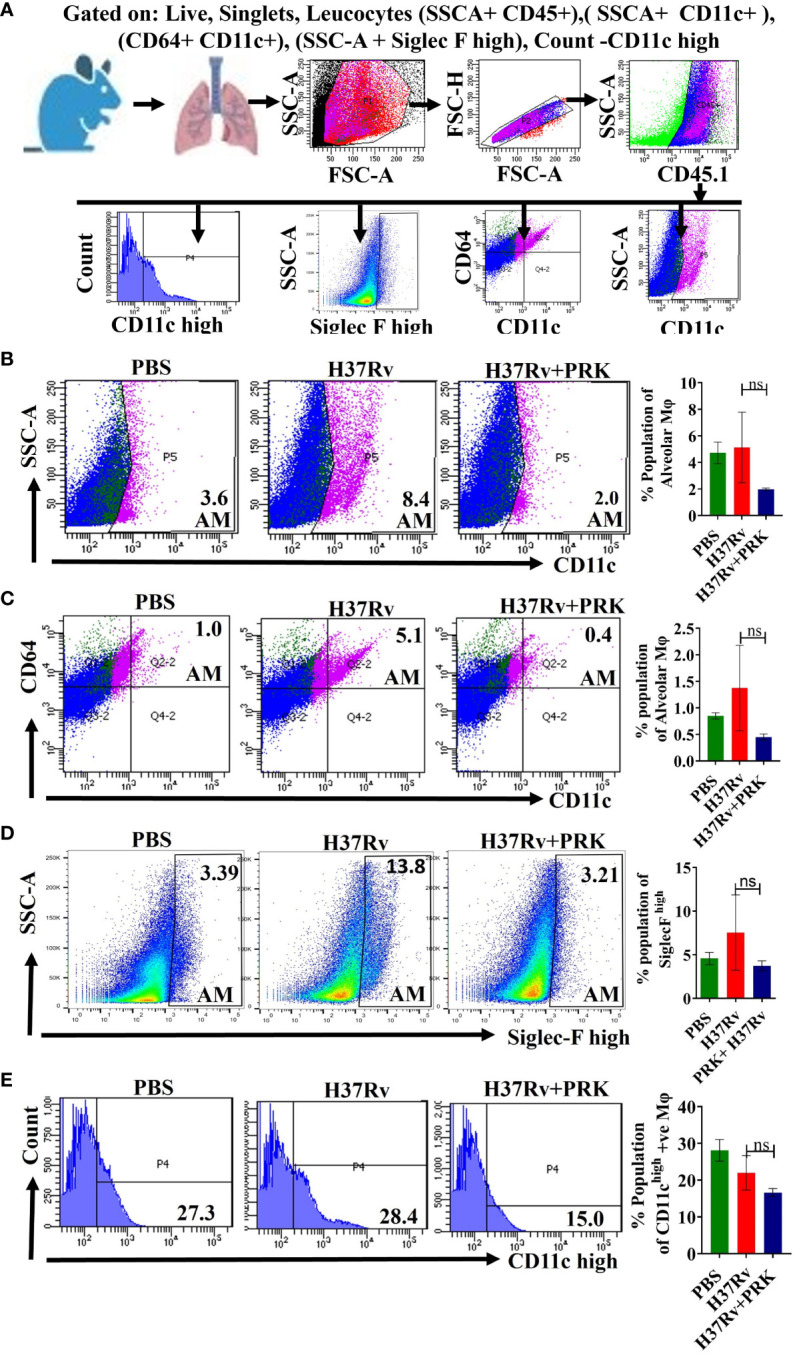
PRK treatment reduced in a somewhat insignificant manner of the alveolar macrophage (AMs) populations in the chronic infection and treatment model. **(A)** General gating strategy used for flow cytometry analysis of lung Immune cells obtained from PBS Control, H37Rv infected, and H37Rv+PRK treated mice (n=4).Identification of lung alveolar macrophages (AMs; SSC-A^+^CD11C^+^ **(B)**; CD64^+^ CD11C^+^ **(C)**, Siglec F^high^ **(D)**, and CD11c^high^ **(E)** macrophage population in the chronic infection and treatment model of mice (harvesting lungs 8 weeks post-infection), showing representative FACS dot plot with the percentage populations (% of parent cells acquired) of macrophages. Data information: Statistical significance between experimental groups was determined by a two-tailed, unpaired Student’s t-test (P > 0.05; n.s. not significant). Mean and standard error (SD) determined from four biological replicates (n = 4) for FACS analysis; Bars indicate means ± SD.

There are two primary types of macrophages found in the lung, the tissue-resident alveolar macrophages (AMs; CD11c^+^, Siglec F^+^), and the interstitial macrophages (IMs; CD11b^+^, Ly6C^+^, and MHC-II^+^), which are derived from monocytes (16, 17, 19, 30). Nevertheless, it has been previously noted that the immune cell population most vulnerable to *Mtb* infection is constituted by permissive monocytes, dendritic cells, and alveolar macrophages (18, 19, 31, 32). Additionally, Huang et al. (19) reported that AMs’ anti-inflammatory properties foster an atmosphere that is favorable for *Mtb* replication and propagation. On the other hand, IMs promote inflammation and regulate the spread and replication of *Mtb*. In the present study, we found that in the acute model of mice infection and treatment, PRK-treated groups had lower populations of double positive, SSC-A^+^CD11c^+^ macrophage phenotypes (**Figures 3A, B**), which are representative of resident alveolar macrophages (AMs), double positive F480^+^Ly6c^+^ monocytes (**Figure 3C**), and dendritic cells (DCs) (**Figure 3D** **).** Because the alveolar macrophage populations rely on the metabolism of fatty acids for energy, they could change into inflammatory foamy macrophages, whose surroundings are favorable for *Mtb* replication and spread (19). The decline in the bacterial load in PRK treatment groups is apparently related to a reduction in the alveolar macrophage population. Similar patterns were also seen in the chronic model of mouse infection, where the populations of macrophages that correspond to alveolar macrophages (AMs); SSC-A^+^CD11c^+^ (**Figures 4A, B**), CD64^+^CD11c^+^ (**Figure 4C**), CD11c^high^ (**Figure 4E**), and SiglecF^high^ (**Figure 4D**) were reduced in a somewhat non-significant manner in the cohort that received PRK treatment. It was earlier reported that macrophages generated from monocytes with CD11c^high^ expression are extremely receptive to *Mtb* infection (**33**) (**Figure 3A**). Taken together, our findings are also consistent with a prior study.

### PRK up-regulates CD11b^+^ IMs populations in acute infection and treatment models of mice

**Figure 5:**
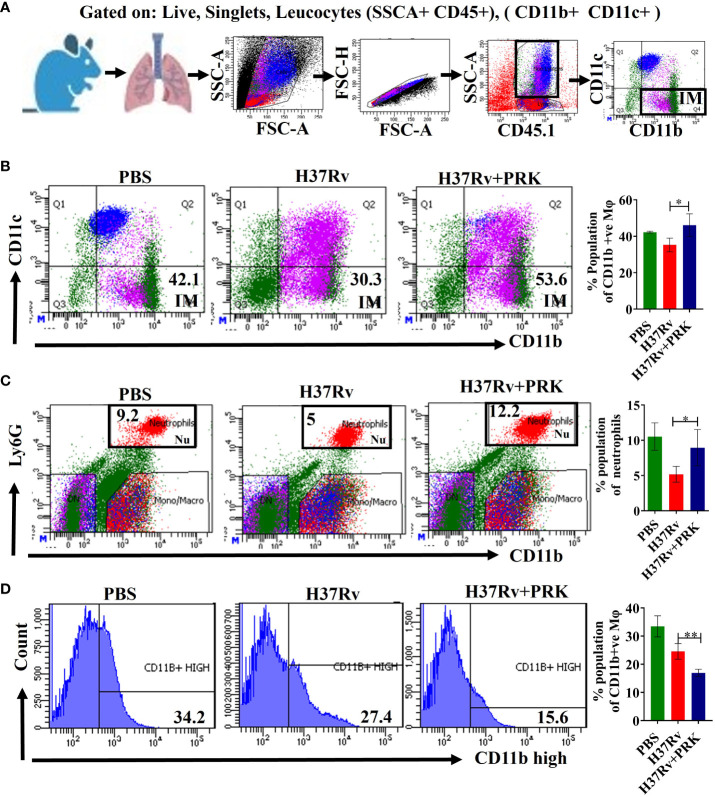
PRK up-regulates CD11b^+^ IMs populations in acute infection and treatment models of mice. **(A)** General gating strategy used for Flow cytometry analysis of lung Immune cells obtained from PBS Control, H37Rv infected, and H37Rv +PRK treated mice (n=4). Identification of lung interstitial macrophages (IMs; CD11b^+^) **(B)** and neutrophils (Ly6G^+^CD11b^+^) **(C)** of the acute infection and treatment model of mice (harvesting lungs 5 weeks post-infection), showing representative FACS dot plot with the percentage populations (% of parent cells acquired) of IMs. **(D)** Identification of lung interstitial macrophages (IMs; CD11b^high)^ of the chronic infection and treatment model of mice (harvesting lungs 8 weeks post-infection), showing representative FACS plot with the percentage populations (% of parent cells acquired) of CD11b^high^ macrophages. Data information: Statistical significance between experimental groups was determined by a two-tailed, unpaired Student’s t-test (*P < 0.05, **P < 0.01). Mean and standard error (SD) determined from four biological replicates (n = 4) for FACS analysis; Bars indicate means ± SD.

The expression of phenotypic markers CD11c and CD11b on macrophages after *Mtb* infection is important in characterizing interstitial macrophages (IMs) (32). Here, we report that the CD11b^+^ (**Figures 5A, B**) and CD11b^+^Ly6G^+^ (**Figure 5C**) macrophage population corresponding to the interstitial macrophage (IMs) and neutrophils, significantly increased in the PRK-treated cohort as compared to the H37Rv infected control in the acute model of infection and treatment. Since interstitial macrophages (IMs) follow glycolytically active pathways, their enrichment is not supportive of the growth of *Mtb* (19). Interestingly, we were not able to observe a similar pattern of interstitial macrophage (CD11b^+^) upregulation in chronic models of infection and treatment, but the CD11b^+^ population significantly reduced in chronic infection and treatment models (**Figure 5D**). Nevertheless, despite the decline in IMs (CD11b^+^) population, we saw a noteworthy decrease in the bacterial load both in the lungs and the spleens in the chronic model of infection and treatment (**Figures 2E, F** **).** Furthermore, Huang et al. (19) reported that depletion of AMs reduced bacterial burden (**Figure 5A**), whereas depletion of IMs increased bacterial burden. On the other hand, we note that post-infection lifetime appears to be a determining factor for both IMs populations and bacterial load, rather than an arithmetic proportion under all settings. IMs population and bacterial burden in the acute model of infection and treatment were shown to be in arithmetic proportion in our experiment; however, in the chronic model of infection and treatment, IMs were not elevated, despite a reduction in bacterial burden. These findings lead us to the conclusion that PRK alters the metabolic transition of host macrophages from fatty acid metabolism to glycolytic metabolism at the log phage of the bacterial infection in the host.

### Pralukast reduced tissue injury by downregulating the Ly6C-positive population

**Figure 6:**
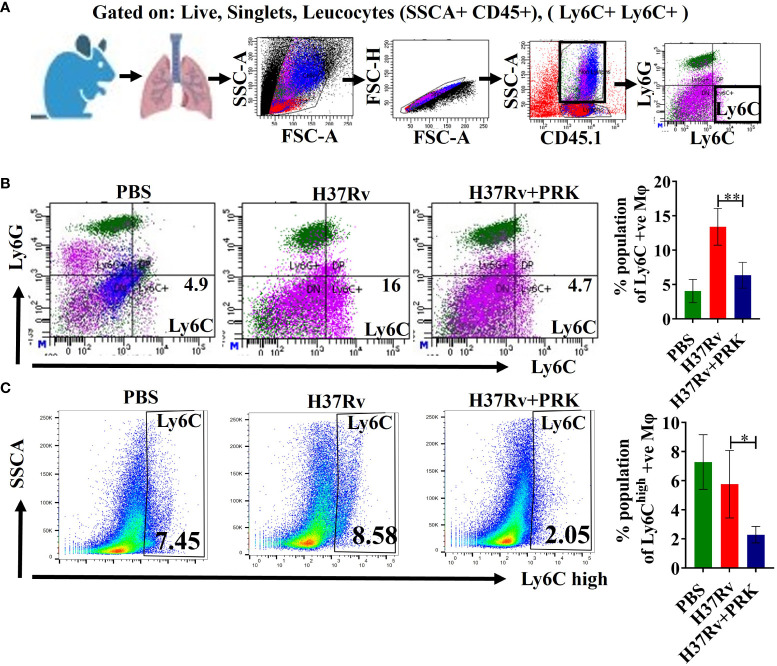
PRK reduced tissue injury by downregulating the Ly6C-positive population. **(A)** General gating strategy used for flow cytometry analysis of lung Immune cells obtained from PBS Control, H37Rv infected, and H37Rv +PRK treated mice (n=4). **(B)** Identification of lung Ly6C^+^ macrophages of the acute infection and treatment model of mice (harvesting lungs 5 weeks post-infection), showing representative FACS dot plot with the percentage populations (% of parent cells acquired) of Ly6C^+^ macrophages. **(C)** Identification of lung Ly6C^high^ macrophages of the chronic infection and treatment model of mice (harvesting lungs 8 weeks post-infection), showing representative FACS plot with the percentage populations (% of parent cells acquired) of Ly6C^high^ macrophages. Data information: Statistical significance between experimental groups was determined by a two-tailed, unpaired Student’s t-test (*P < 0.05, **P < 0.01). Mean and standard error (SD) determined from four biological replicates (n = 4) for FACS analysis; Bars indicate means ± SD.

The expression of the TREM-2 marker on Ly6c^+^ macrophages determine their activity in a proportional manner (34, 35). Furthermore, *Mtb*-infected mice showed monocytosis at 2- and 4 weeks post-infection, as evidenced by a rise in Ly6C^high^, CD11b^+^, and CD115^+^ cells in the blood (19). Normally, in the acute phase of tissue damage, Ly6C^high^ inflammatory monocyte precursors move to wounded areas and cause inflammation. Nonetheless, monocyte-derived macrophages aid in the resolution of inflammation and tissue healing if the sources of tissue injury are removed (36, 37) Consistent with the above, we observed an upregulation of Ly6C^+^ and Ly6C^high^ inflammatory monocytes after 5 and 8 weeks of post-infection in H37Rv control groups of acute and chronic infection model, respectively; into contrast, we saw a notable decrease in Ly6C^+^ and Ly6C^high^ inflammatory monocytes in the groups that received PRK treatment in both acute and chronic model of infection and treatment (**Figures 6A–C**).

### PRK stimulates Ym1+Ym2 and Arginase1-positive populations of lung macrophage to heal lung pathology

**Figure 7:**
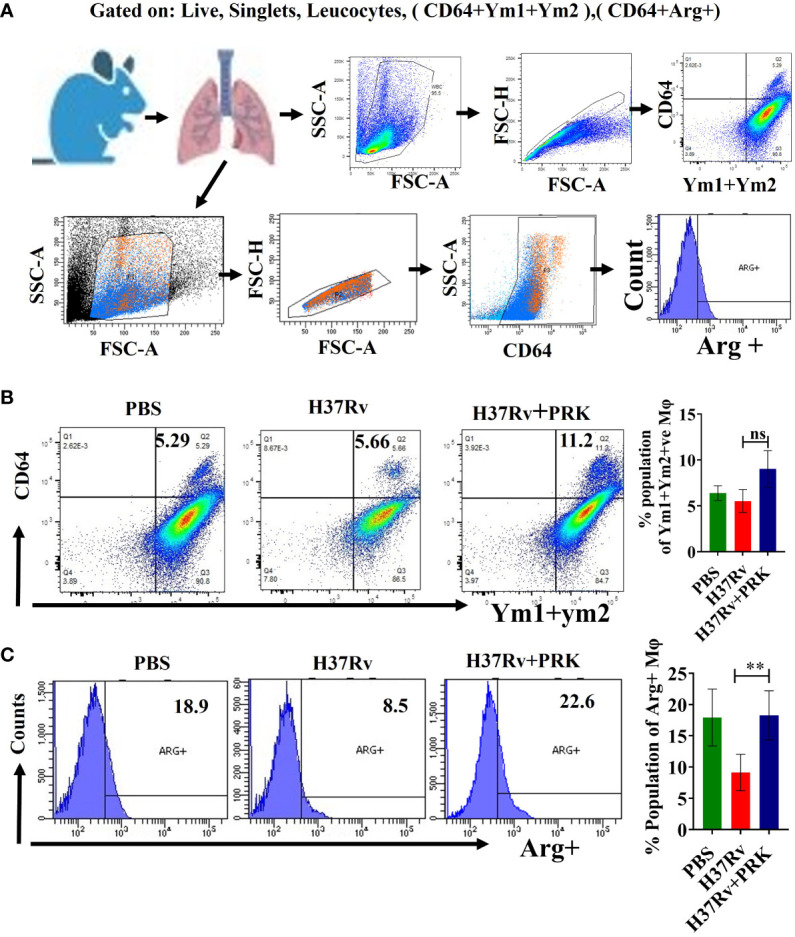
PRK stimulates Ym1+Ym2 and Arginase1-positive populations of lung macrophage to heal lung pathology **(A)** General gating strategy used for flow cytometry analysis of lung Immune cells obtained from PBS Control, H37Rv infected, and H37Rv +PRK treated mice. **(B)** Identification of lung Ym1+Ym2 ^+^(chitinase-3 like 3) macrophages of the chronic infection and treatment model of mice (harvesting lungs 8 weeks post-infection), showing representative FACS dot plot with the percentage populations (% of parent cells acquired) of Ym1+Ym2 ^+^ macrophages (n=3) **(C)** Identification of the lung arginase-1 (Arg-1), Arg^+^ macrophages of the chronic infection and treatment model of mice (harvesting lungs 8 weeks post-infection), showing representative FACS plot with the percentage populations (% of parent cells acquired) of Arg^+^ macrophages (n=4). Data information: Statistical significance between experimental groups was determined by a two-tailed, unpaired Student’s t-test (**P < 0.01, and n.s. not significant). Mean and standard error (SD) determined from three and four biological replicates (n = 3/4) for FACS analysis; Bars indicate means ± SD.

Microbial infection results in tissue damage and acute inflammation by the activation and assembly of multiple immune cell types at the injury site. Additionally, following the elimination of the tissue injury’s source, the activated and assembled immune cells coordinate the reduction of inflammation, tissue repair, and regeneration (30, 35, 38–40). The primary movers in tissue regeneration and repair following microbial infection are tissue-resident macrophages and inflammatory monocytes. They achieve this by phenotypically altering their recognition and activity during the resolution phases of inflammation and injury (14, 35). In mice, the expression of the proteins Ym1 (chitinase 3-like 3) and arginase-1 (Arg-1) consistently indicates the presence of tissue repair-associated macrophage phenotypes that are variable in activation (15, 37, 38, 41–43). Here, we report that, in the chronic model of infection and therapy, PRK-treated mice showed an increase in the Ym1+Ym2 (**Figures 7A, B**) and arginase-1 (Arg-1) (**Figure 7C**) positive population of macrophage in comparison to the H37Rv infected group. Since Arginase -1 (Arg-1) and Ym1+Ym2 positive macrophages are associated with the healing of lung tissues, our observation of their upregulation highlights a salutary role of PRK not only in reducing the tubercular burden but also in the subsequent healing of the tissue pathology. Given that the repair of lung tissues is linked to Arginase-1 (Arg-1) and Ym1+Ym2 positive macrophages, our observation of their upregulation underscores a protective function of PRK in both lowering the tubercular burden and promoting the healing of tissue disease that follows. The results of immune response in acute and chronic infection model are now summarized in **Table 3**. (**Supplementary material; Supplementary Figure 1**).

**Table 3:** Immune response in PRK treated group as compared to H37Rv control group.

Infection and treatment model	AMs (SSC-A^+^CD11c^+)^	IMs (CD11b^+^)	Ly6C^+^ macrophage	Arginase1^+^
Chronic	↓ Non-significantly	↓ Significantly	↓ Significantly	↑ Significantly
Acute	↓ Significantly	↑ Significantly	↓ Significantly	DNP

↓(downregulated); ↑(upregulated); DNP (data not presented).

## Discussion

We previously reported on the direct killing impact of pranlukast (PRK) on *Mtb* by the allosteric inhibition of *Mtb* ornithine acetyltransferase, a crucial enzyme in the arginine biosynthesis pathway resulting in the inhibition of arginine production in the bacteria (26). In the current study, we examined the host immune effectors that lower the bacterial burden and promote lung healing in PRK-treated mice. We carried out the infection and treatment experiment to examine the activity of PRK in acute as well as chronic *Mtb* infection models of mice after establishing that PRK shows no detrimental influence on mice’s health and no toxicity on the essential organs such as the liver, kidney, and lungs. We found that PRK functions more effectively during the acute stage of bacterial infection than it does during the chronic stage. The CFU count has a strong correlation with the macroscopic pathology and histopathology of the infected lungs.

Furthermore, in the acute model of mice infection, we found that PRK-treated groups significantly downregulated CD11c^+^ macrophage phenotypes corresponding to resident alveolar macrophage (AMs) and dendritic cells (DCs), as well as monocyte populations. A decline in the alveolar macrophage population may be the cause of the bacterial load reduction in PRK treatment groups since these macrophages are dependent on fatty acid metabolism and can develop into pro-inflammatory foamy macrophages, whose milieu is favorable for *Mtb* growth and dissemination. A comparable result was also noted in the chronic model of mouse infection, where we found that the PRK-treated group had down-regulated populations of CD11c^+^, CD64^+^CD11c^+^, CD11c^high^, and Siglec F^high^, which correlate to alveolar macrophages.

Furthermore, we found that in the acute model of infection and treatment, the PRK-treated group exhibited a significant increase in the CD11b^+^ and CD11b^+^Ly6G^+^ macrophage population, which corresponds to interstitial macrophages (IMs) and neutrophils, as compared to the H37Rv infected control. Interstitial macrophage (IMs) enrichment is detrimental to *Mtb* proliferation because IMs adhere to glycolytically active pathways (19, 44). However, CD11b^+^ populations were dramatically reduced in the chronic infection and treatment model, which is interesting because we were not able to find a comparable pattern of Interstitial macrophage (CD11b^+^) upregulation in these models. However, in the chronic model of infection and therapy, we observed a significant decrease in the bacterial burden despite the decline in IMs (CD11b^+^) population. Moreover, Huang et al. (19) found that whereas IM depletion increased bacterial burden, AM depletion decreased it. However, rather than being an arithmetic proportion in all cases, we see that post-infection lifetime seems to be a determining factor for both IMs populations and bacterial load. In our experiment, we found that in the acute model of infection and treatment, IMs populations and bacterial burden were in arithmetic proportion; in contrast, despite a decrease in bacterial burden, IMs were not enhanced in the chronic model of infection and treatment.

After analyzing these data, we may draw the conclusion that PRK causes the host macrophages to switch from fatty acid metabolism to glycolytic metabolism in the vicinity of the log phage bacterial infection. Since blood monocytes are the source of IMs and *Mtb* infection upregulates blood monocytes (Ly6c^high^), monocytosis results, which is dependent on the quantity of infecting bacteria. In the current study, the day 1 bacterial infection in the chronic mode of infection and treatment was lower (100 CFU) than the acute infection model (500 CFU). However, after 4 weeks of infection, the former has a lower bacterial load than the acute model of infection, and the bacterial load further decreases with PRK treatment, leading to a reduction in monocytosis. As a result, there were fewer IMs in the chronic infection and therapy mode. This is in line with earlier studies showing that lung macrophage population alteration can have a significant impact on bacterial load, both favorably and unfavorably (45, 46). Furthermore, these earlier investigations suggest that lung macrophages’ permissiveness to bacterial growth may play a role in the course of the disease in addition to immunological control. The inflammatory response is first and foremost essential for the removal of germs. However, the initial inflammatory response is probably switched to tissue repair and regeneration processes if the sources of tissue injury are removed (14, 35). Nevertheless, according to (Murray & Wynn (47), macrophages are essential for both the development and remission of inflammation. Tissue-resident macrophages and invading monocytes jointly cause inflammation during the acute inflammatory phase by secreting inflammatory cytokines and chemokines. On the other hand, during the phase of inflammatory recovery, these cells also support tissue regeneration and repair (14, 19). Further, Huang et al. (19) reported that *Mtb*-infected mice developed monocytosis, as evidenced by increased numbers of Ly6C^high^ CD11b^+^ CD115^+^ cells in the blood at 2- and 4 weeks post-infection. In the acute phase of tissue damage, Ly6C^high^ inflammatory monocyte precursors normally move to wounded areas and cause inflammation. However, monocyte-derived macrophages aid in the resolution of inflammation and tissue healing if the underlying causes of tissue damage are removed (36, 37). Here we document a comparable pattern of elevation of Ly6C^high^ inflammatory monocyte following 5 and 8 weeks of *Mtb* post-infection in H37Rv control groups, while a notable decrease in Ly6C^+^ inflammatory monocyte was noted in the groups treated with PRK. Consistent with the above findings, we first saw an increase in the populations of proinflammatory macrophages (IMs) in the PRK-treated groups in the acute model of infection. However, in the chronic model of mice infection, the Ym1+Ym2 and Arg-1-positive population of macrophage increased in PRK-treated mice as compared to the H37Rv-infected control group. We believe that the emergence of Ym1+Ym2 and Arg-1 positive subpopulation of lung macrophage contributes to the resolution of inflammation and tissue repair in the PRK treatment group as compared to the untreated infected controls. This is reminiscent of the role of the expression of Arg-1 positive populations of macrophages in the wound-healing process as reported by (37, 48). Our findings are consistent with previous research demonstrating that manipulation of the lung macrophage population can have a significant impact on the bacterial burden (45, 46). PRK not only lowers the tubercular load but also augments the tissue repair process consequently, it augurs well for induction in anti-tubercular therapeutics.

## Data availability statement

The original contributions presented in the study are included in the article/**Supplementary Material**. Further inquiries can be directed to the corresponding authors.

## Ethics statement

The animal study was approved by Institute Animal Ethics Committee- CAF/ETHICS/899/2022. The study was conducted in accordance with the local legislation and institutional requirements.

## Author contributions

RR: Conceptualization, Data curation, Formal analysis, Investigation, Methodology, Supervision, Validation, Visualization, Writing – original draft, Writing – review & editing. AS: Conceptualization, Funding acquisition, Project administration, Resources, Writing – review & editing.
