# Supplementary material for: Antimycobacterial and healing effects of Pranlukast against MTB infection and pathogenesis in a preclinical mouse model of tuberculosis
Source: Front Immunol. 2024 May 2;15:1347045. doi: 10.3389/fimmu.2024.1347045 (PMC11096513; doi:10.3389/fimmu.2024.1347045)
Supplement: Supplementary file 1 [file DataSheet_1.docx]

**The killing and healing effects of Pranlukast (PRK) against MTB infection and pathogenesis in a preclinical mouse model of tuberculosis.**

**Authors: RS Rajmani^1*^ and Avadhesha Surolia^1*^**

**Affiliations:**

^1^Molecular Biophysics Unit, Indian Institute of Science, Bangalore-560012

*Corresponding authors; E-mail: raju.raj.rajmani5@gmail.com [surolia@iisc.ac.in](mailto:surolia@iisc.ac.in)

**Supplementary Table-1**

| S.No. | Antibodies | Catalog no. and company |
| --- | --- | --- |
| 1. | Alexa Fluor® 700 Mouse Anti-Mouse CD45.1 | 561235  BD Pharmingen™ |
| 2. | APC-Cy™7 Rat Anti-Mouse CD19 | 557655  BD Pharmingen™ |
| 3. | PE-Cy™7 Hamster Anti-Mouse CD11c | 558079  BD Pharmingen™ |
| 4. | BUV395 Hamster Anti-Mouse CD11c | 564080  BD Horizon™ |
| 5. | BV480 Rat Anti-CD11b | 566117  BD Horizon™ |
| 6. | BV510 Rat Anti-Mouse Ly-6G | 740157  BD OptiBuild™ |
| 7. | BV605 Rat Anti-Mouse Ly-6C | 563011  BD Horizon™ |
| 8. | APC Rat Anti-Mouse Ly-6G and Ly-6C | 561083  BD Pharmingen™ |
| 9. | Alexa Fluor® 488 Rat Anti-Mouse F4/80-Like Receptor | 564227  BD Pharmingen™ |
| 10. | Alexa Fluor® 647 Mouse anti-Mouse CD64 a and b Alloantigens | 558539  BD Pharmingen™ |
| 11. | BV711 Rat Anti-Mouse Mer | 747892  BD OptiBuild™ |
| 12. | PerCP-Cy™5.5 Rat Anti-Mouse Siglec-F | 565526  BD Pharmingen™ |
| 13. | Arginase 1 Monoclonal Antibody (A1exF5), eFluor 450 | # 48-3697-82  eBioscience™ |
| 14. | PE Anti-Ym-1 + Ym-2 antibody [EPR15263] | ab211621  Abcam |
| 15. | Purified Rat Anti-Mouse CD16/CD32 (Mouse BD Fc Block™) BD Pharmingen™ | 553141  BD Pharmingen™ |
| 16. | BV650 Rat Anti-Mouse I-A/I-E(MHC-II) | 563415  BD Horizon™ |
|  |  |  |


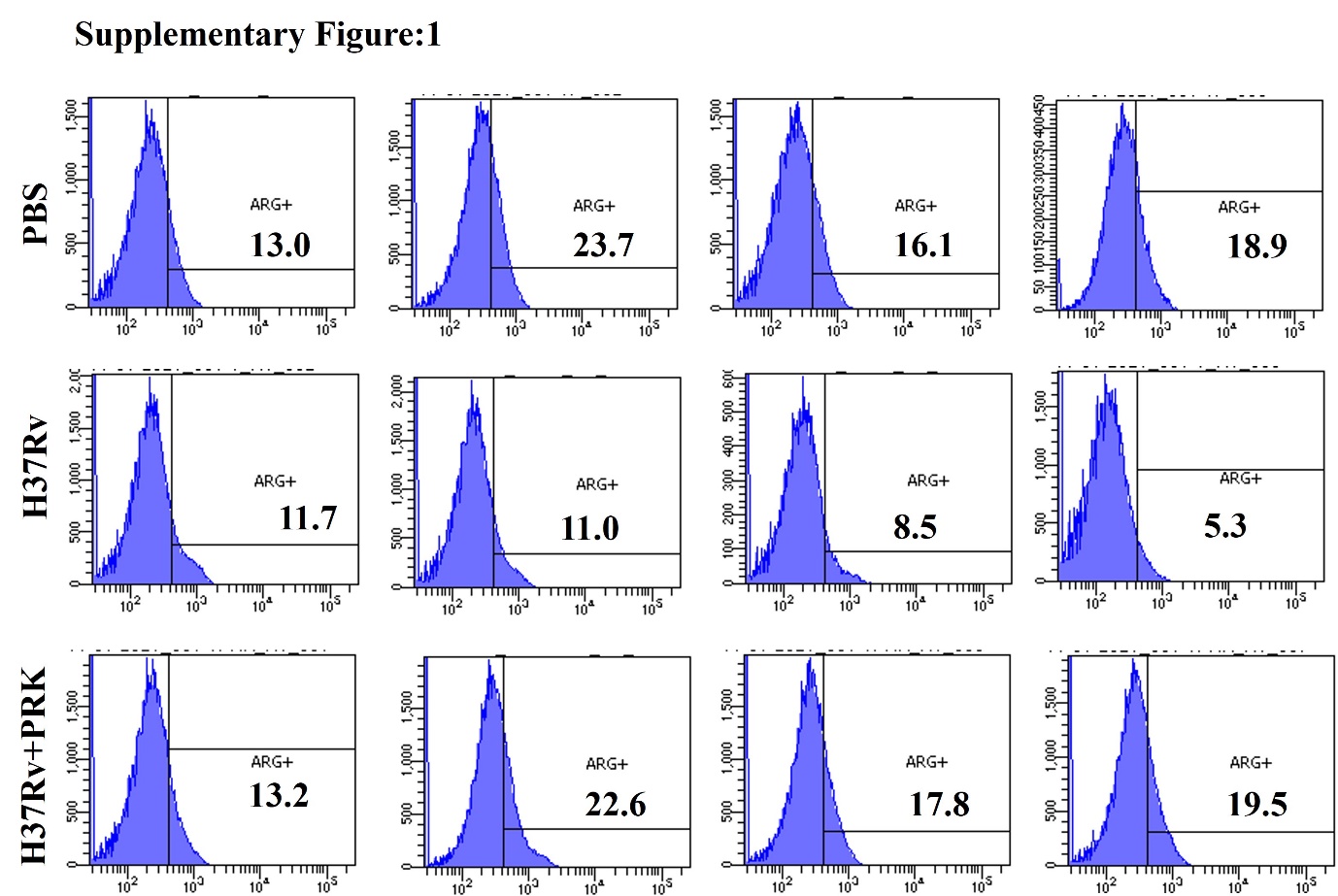


**Supplementary Figure-1:** Identification of lung arginase-1 (Arg-1), Arg^+^ macrophages of the chronic infection and treatment model of mice (harvesting lungs 8 weeks post-infection), showing representative FACS plot with the percentage populations (% of parent cells acquired) of Arg^+^ macrophages(n=4).
